# The relevance of clinical ethnography: reflections on 10 years of a cultural consultation service

**DOI:** 10.1186/s12913-017-2823-x

**Published:** 2018-01-11

**Authors:** Melissa Dominicé Dao, Sophie Inglin, Sarah Vilpert, Patricia Hudelson

**Affiliations:** 1 Department of Community Medicine, Primary Care and Emergency Medicine, Geneva University Hospitals, 4 rue Gabrielle-Perret-Gentil, 1211 Geneva, Switzerland; 2 Institute of Primary Care Medicine, Faculty of Medicine, University of Geneva, Geneva, Switzerland; 3 Geneva, Switzerland; 4 Institute of social and preventive medicine, University of Lausanne, Lausanne, Switzerland

**Keywords:** Cultural consultation, Clinical ethnography, Cultural formulation, Intercultural communication

## Abstract

**Background:**

Training health professionals in culturally sensitive medical interviewing has been widely promoted as a strategy for improving intercultural communication and for helping clinicians to consider patients’ social and cultural contexts and improve patient outcomes. Clinical ethnography encourages clinicians to explore the patient’s explanatory model of illness, recourse to traditional and alternative healing practices, healthcare expectations and social context, and to use this information to negotiate a mutually acceptable treatment plan. However, while clinical ethnographic interviewing skills can be successfully taught and learned, the “real-world” context of medical practice may impose barriers to such patient-centered interviewing. Creating opportunities for role modeling and critical reflection may help overcome some of these barriers, and contribute to improved intercultural communication in healthcare.

We report and reflect on a retrospective analysis of 10 years experience with a “cultural consultation service” (CCS) whose aim is to provide direct support to clinicians who encounter intercultural difficulties and to model the usefulness of clinical ethnographic interviewing for patient care.

**Methods:**

We analyzed 236 cultural consultation requests in order to identify key patient, provider and consultation characteristics, as well as the cross cultural communication challenges that motivate health care professionals to request a cultural consultation. In addition, we interviewed 51 clinicians about their experience and satisfaction with the CCS.

**Results:**

Requests for cultural consultations tended to involve patient care situations with complex social, cultural and medical issues. All patients had a migration background, two-thirds spoke French less than fluently. In over half the cases, patients had a high degree of social vulnerability, compromising illness management. Effective communication was hindered by language barriers and undetected or underestimated patient/provider differences in health-related knowledge and beliefs. Clinicians were highly satisfied with the CCS, and appreciated both the opportunity to observe how clinical ethnographic interviewing is done and the increased knowledge they gained of their patients’ context and perspective.

**Conclusions:**

A cultural consultation service such as ours can contribute to institutional cultural competence by drawing attention to the challenges of caring for diverse patient populations, identifying the training needs of clinicians and gaps in resource provision, and providing hands-on experience with clinical ethnographic interviewing.

## Background

Providing health care across social, cultural and linguistic differences is challenging and may lead to health care disparities and lower levels of care [1]. Difficulties can arise from patient-provider differences in language, communication styles, health-related knowledge, values, expectations and behaviors, and from providers’ inability to identify and take into account these differences when caring for patients [2–5].

Training health professionals in culturally sensitive, medical interviewing has been widely promoted as a strategy for improving intercultural communication and helping clinicians to consider patients’ social and cultural contexts [6–8]. A number of patient-centered interviewing models have been developed that integrate cultural factors into the biopsychosocial model [9–13]. These approaches encourage clinicians to explore the patient’s explanatory model of illness, recourse to traditional and alternative healing practices, healthcare expectations and social context, and to use this information to negotiate a mutually acceptable treatment plan. Through clinical ethnography, “the clinician can empathize with the lived experience of the patient's illness, and try to understand the illness as the patient understands, feels, perceives, and responds to it.” [8] Training in such approaches can lead to improved patient outcomes [14].

However, while clinical ethnographic interviewing skills can be successfully taught and learned [15, 16], some studies suggest that in the context of “real-world” medical practice, overworked clinicians give minimal attention to patient-centered interviewing [17, 18]. Clinicians may be reluctant to explore-- and unprepared to address-- patients’ psychosocial problems [19, 20]. Time pressures, an emphasis on constructing case histories void of “extraneous” information, and an unspoken assumption that medicine is culturally neutral can hinder exploration of and attention to social and cultural aspects of care [21–25]. Some authors have argued for role modeling and critical reflection in order to counter the potentially negative effects of training on clinicians’ attitudes towards the care of socially and culturally diverse patients [26, 27].

In this paper, we report on a retrospective analysis of 10 years experience with a “cultural consultation service” (CCS) whose aim is to provide direct support to clinicians who encounter intercultural difficulties and to model the usefulness of clinical ethnographic interviewing for patient care. We describe the cross cultural communication challenges that motivate health care professionals to contact the CCS, their satisfaction with the support we provide, and conclude with a discussion of the role of cultural consultations as part of an integrated strategy to build institutional capacity to provide quality care for diverse populations.

### Geneva university hospitals

The Geneva University Hospitals (Hôpitaux Universitaires de Genève, or HUG) is an 1800-bed hospital group serving a diverse population. Forty percent of Geneva residents are of foreign nationality (190 nationalities) and 20% of Geneva residents speak a language other than French as their primary language [28]. At the HUG, about half of patients and half of the staff are of foreign nationality and one in 12 patients speak no French at all [29].

In this context of “hyper-diversity” [30], health professionals at the HUG regularly care for patients who differ from them in terms of language, education and social context, and frequently encounter challenges such as language barriers, difficulty understanding patients’ complaints, illness-related beliefs that contradict medical knowledge, patients’ social problems they feel ill-equipped to deal with, and lack of time to adequately address patients’ needs [31–33]. In a survey of medical students and doctors at the HUG, respondents rated themselves least competent at intercultural communication skills in situations with the greatest social and cultural differences between patient and doctor: undocumented immigrants, asylum seekers, and patients with illness-related beliefs at odds with biomedicine [34].

### The cultural consultation

In 2006 we created a cultural consultation service (CCS) to provide direct support to HUG clinicians who encountered cross-cultural communication difficulties [35]. We announced the CCS at new-staff orientation and continuing education seminars, and created a hospital webpage with information about the CCS and how to contact us.

Our CCS is modelled on the original CCS first developed by Kirmayer and colleagues for mental health practitioners in Montreal [36]. However, our CCS is located in the Division of Primary Care and accepts cultural consultation (CC) requests from all hospital departments; there is no specific focus on mental health care.

Assessments are conducted by one of two CCS consultants, a general internal medicine attending physician (MDD) and a medical anthropologist (PH), both of whom actively participate in a number of teaching activities aimed at strengthening health professionals’ intercultural competence.^1^

For each CC request, an “intake form” is filled with basic information about the request and the referring clinician. Occasionally the cultural consultant may discuss the case with the referring clinician or team and provide suggestions without seeing the patient directly. In these cases, the consultant makes note of the discussion and recommendations on the intake form.

In the majority of cases, the CCS consultant meets with the patient (often several times) to explore the social and cultural factors influencing communication and care. The referring clinician is invited to participate in the patient interview when possible. Interviews are conducted in the patient’s preferred language through an interpreter, or in French or English when the patient demonstrates adequate fluency.

To guide our patient interviews, we created a modified version of the DSM-IV Outline for Cultural Formulation (CF) [37, 38]. The Outline for Cultural Formation provides a framework for collecting clinically relevant social and cultural information, and is used to elicit patients’ illness experiences and consider their illness in social context [39]. Like the original CF, our interview guide includes the patient’s social and cultural identity, the patient’s explanatory model of the current illness, social factors affecting communication and access to care; and factors affecting the patient/provider relationship. However, we adapted the Cultural Formation Outline to include a more explicit focus on migration-related and social factors. Table 1 outlines the main categories driving information gathering. Not all categories of information were necessarily collected for all patients, nor were topics addressed in any particular order. Rather, interviews were conducted as unstructured, narrative conversations with patients, who were encouraged to talk about what most concerned them. Cultural consultants then probed for further details on issues that arose.

**Table 1:** Modified cultural formulation guide used in cultural assessments

Patient’s cultural identity	• Cultural reference group(s) • Languages spoken • Religion/spirituality • Schooling, professional experience • Migration history • Level/type of integration (with cultures of home and host countries)
Patient’s social context	• Work, income, legal status • Sources of stress • Sources of support and health information • Social networks and activities • Impact of illness on social functioning
Patient’s explanatory model	• Main complaint • Meaning, explanation and perceived severity of current illness • Treatment–seeking for current illness • Previous illness and health care experiences (self and others) • Treatment expectations • Worries, concerns, priorities with regards to current illness
Provider/patient relationship	• Patient/provider ethnic, social and cultural differences • Value conflicts • Possible sources of prejudice and bias (of patient and provider) • Differences in understanding of medicine and the health care system

Immediate results of the assessment are usually transmitted back to the referring clinician via a brief email and/or a phone call. A subsequent detailed consultation report is prepared for the clinician, including recommendations for overcoming communication difficulties encountered by the referring clinician. This report is inserted in the patient’s electronic health record and accessible by all hospital clinicians.

We discuss CC requests monthly with an expanded CCS group, consisting of primary care physicians, psychiatrists, a nurse and a psychologist who are trained and experienced in intercultural medicine. Referring clinicians are invited to participate in the CCS group discussion of their case. The purpose of these discussions is to invite additional perspectives and suggestions for understanding and overcoming the difficulties that motivated the CC request.

## Methods

We reviewed all requests and all CC patient assessment reports from March 2006 to December 2015. Intake forms (completed for each CC request, and containing basic information about the patient, clinician, and the CC request) and full CC reports were reviewed to identify key characteristics of each request (Table 2).

**Table 2:** Key characteristics of cultural consultation case requests

Referring clinician’s characteristics	• Hospital department/division • Type of professional • Function
Patient characteristics	• Age and sex • Nationality and type of permit • Years spent in Switzerland • Religion • Language abilities • Level of education • Main diagnosis
Cultural consultation characteristics	• The nature of the referring clinician’s request to the CCS • Whether or not a patient assessment was conducted • Presence of an interpreter or a cultural informant during the assessment • Key issues affecting care that were identified during the assessment • Recommendations made to the referring clinician

A coding scheme was developed for each of the categories above. The authors first read through several CC reports and intake forms, and created tentative codes for each of the categories. The codes were then tested on a new set of CCs, and new or modified codes were created as necessary. The authors then independently coded all CCs, and compared/discussed their coding to resolve any discrepancies.

In addition, all 76 clinicians who had requested a consultation between March 2006 and October 2008 and between September 2009 and October 2011 were invited to participate in a short interview conducted by SV and SI (independent research assistants not involved in the CCs). For these interviews we developed a series of structured questions based on the evaluation questionnaire used by the Montreal CCS [40] (Table 3). Open-ended responses and spontaneous comments were noted down verbatim by the interviewers. Interviews were generally conducted about 1 month after the CC report was sent to them. Frequencies were calculated for answers to questions 1–6 using SPSS Version 22. SI and SV organized spontaneous comments and open-ended by theme, which were read and verified by MDD.

**Table 3:** Evaluation questionnaire for clinicians requesting a cultural consultation

Question	Type of answer
How satisfied were you with the CC?	6 point Likert scale, from “not at all satisfied” to “perfectly satisfied”
How useful was the CC?	6 point Likert scale, from “not at all useful” to “extremely useful”
Would you recommend the CCS to your colleagues?	Yes/No
Would you request a CC in the future if needed?	Yes/No
How important to you are the following aspects of the CCS? • Sociocultural expertise of the consultant • A time and space for discussing complex cases • The perspective of an outside consultant	Very/Somewhat/Not at all (one answer per item)
How did the CC help you? • Better understand the patient’s illness-related ideas and expectations • Communicate more effectively with the patient • Clarify the patient’s diagnosis • Clarify the patient’s treatment plan • Improve the patient’s adherence to treatment • Better understand how social and cultural factors affect the case • Better understand asylum and/or immigration related issues • Learn about community resources available for immigrant patients	Yes/No/Not applicable (one answer per item)
What suggestions do you have for improving the CCS?	Open-ended question
Do you have any other comments you would like to add?	Open-ended question

## Results

### Characteristics of CC requests

Between March 2006 and December 2015, we received 236 CC requests, with an average of about 2–3 per month. A majority of requests came from physicians working in general internal medicine, with a small majority of hospitalized patients (Table 4).

**Table 4:** Characteristics of referring clinician (*n* = 236)

Characteristic	*N*	%
Hospital department of referring clinician		
General/internal medicine	135	57.2
Paediatrics	36	15.2
Psychiatry	19	8.1
Other	46	19.5
Type of care provided to referred patient at time of consultation		
Inpatient	132	55.9
Outpatient	100	42.3
Mixed case	4	1.7
Profession of referring clinician		
Physician	198	83.9
*Resident*	*125*	*53.0*
*Attending*	*59*	*25.0*
*Independent practice*	*14*	*5.9*
Nurse	28	11.9
Social worker/psychologist	9	4.2

50.4% of referred patients were female. Ages ranged from 1 to 97 years with a median age of 37. All patients were either first (213; 90.2%) or second generation (23; 9.8%) migrants with a very high proportion of African background (Table 5). 30.9% of patients had completed secondary school or attended university. Patients’ health problems were mainly chronic diseases, often presenting at a severe stage.

**Table 5:** Characteristics of referred patients (*N* = 236)

Patient characteristic	*N*	%
Patient’s proficiency in French		
Fluent in French	79	33.5
Basic knowledge	84	35.5
No French spoken	73	31.0
Region of origin		
Africa	134	56.8
Subsaharan Africa	94	39.8
East Africa	32	13.6
North Africa	8	3.4
Asia	50	21.4
Indian subcontinent and Sri Lanka	19	8.1
Middle-east	13	5.5
South-east Asia	11	4.7
Other	7	3.0
Europe	42	17.9
Balkans	35	14.8
Other	7	3.0
America	8	3.4
Central America	5	2.1
South America	3	1.3
Missing data	2	0.8
Migration status in Switzerland		
Stable residency permit/Swiss passport	111	47.0
Asylum seeker/undocumented migrant	79	33.5
Other (tourist, diplomat, etc.)	9	3.8
Missing data	37	15.7
Time spent in Switzerland		
Less than 1 year	46	19.5
1 to 10 years	68	28.8
More than 10 years	79	33.5
Missing data	43	18.2
Religion		
Muslim	77	32.6
Christian	75	31.8
Other	12	5.1
Missing data	72	30.5
Education		
None	23	9.7
Primary school completed	29	12.3
Secondary school completed	46	19.5
University degree	27	11.5
Missing data	111	47.0
Main medical problem		
Infectious disease (HIV, TB)	34	14.4
Chronic pain/somatization	28	11.9
Mental health problem	27	11.4
Cancer	21	8.9
Neurological disease	18	7.6
Other	108	45.8

### Reasons for CC requests

When contacting the CCS, clinicians’ usually began by describing the particular clinical difficulty they were having, such as the patient’s non-adherence to treatment recommendations, uncertainty about the patient’s diagnosis or doubts about the patient’s understanding of his/her illness and treatment. They were often uncertain as to how culture might be affecting the situation, but thought “something cultural” was going on. When asked more precisely how they thought the CCS could help, they would usually evoke a desire to better understand the patient’s illness-related beliefs and social context. Occasionally clinicians’ requests were quite specific, such as help in managing a conflict with the patient, while in others the request was for general information on the cultural aspects of a particular health-related issue (violence, TB, HIV, etc.) (Table 6).

**Table 6:** Categories of requests made by the referring clinicians to the Cultural consultation service (CCS)

Category of request addressed to the CCS	*N* ^a^	%
Help resolve specific clinical issue		
Improve patient’s treatment adherence	86	36.4
Evaluate patient’s diagnosis	27	11.4
Verify the patient’s illness comprehension and ability to give informed consent	22	9.3
Improve general understanding of the patient		
Clarify patient’s illness-related beliefs and practices	76	32.2
Provide information about the patient’s social situation and living conditions	116	49.2
Request for general information about a religious or ethnic community	81	34.3
Clarify expectations of patient and/or family	20	8.5
Other	81	34.3

^a^The sum of requests is greater than 236 because clinicians often formulated multiple requests

We were not able to find patterns among the multiple types of requests made by clinicians for these situations with multiple layers of complexity.

Table 7 provides text examples of typical CC requests. In these three examples, a number of patient characteristics have been removed to protect their anonymity.

**Table 7:** Examples of cultural consultation requests

Brief clinical description	Requests made by the referring clinician
A. Young recent immigrant female patient, illiterate and with very basic French language ability who was recently diagnosed with sarcoidosis. The patient complains of drug side effects (despite low-dose treatment), massive weight gain and chronic pain. The patient is depressed and hides her illness from her family and community.	Her physician would like to better understand why her illness is viewed so negatively by the patient and her family/community.
B. Female visible minority patient in her late twenties, hospitalized for 3 weeks for an acute abdominal infection. Treated unsuccessfully with antibiotics and a drain, she is now refusing all treatments and wants to leave the hospital. When her doctor explained that this would lead to serious consequences for her health, the patient and her mother became angry, stating that only God could predict the future.	Her physician would like help in overcoming this conflict so that he can treat the patient efficiently.
C. Middle aged male ex-refugee patient suffering from chronic pain and disability of the shoulder after an accident 10 years earlier, which was followed by significant social decline. He also presented with anxiety and obsessive-compulsive disorder (OCD) with no improvement despite medical treatment and psychotherapy.	The patient’s family doctor and psychologist referred the patient because they wished to better understand his migration history. Also they were puzzled by the cultural aspects of his obsessive thoughts (karmic interpretation of misfortune) and were uncertain how to help the patient.

### Results of the CCS assessment

One hundred and-fifty of the 236 CC requests (63.6%) resulted in a patient assessment, while in 45 cases (19.0%) we provided only over-the-phone advice and information to the requesting clinician. In 16 cases (7%), we provided a clinical supervision with the referring clinician or team in the absence of the patient. We provided no response in 25 cases (10.5%) either because the problem resolved without our help, the patient was discharged, or because a cultural consultant was unavailable at that time.

#### Factors affecting communication and care

The social and cultural factors affecting care as identified by the CCS and noted in either the intake forms or patient assessment reports are listed in Table 8. In most cases, we identified several factors affecting communication and care.

**Table 8:** Issues identified during the cultural assessment (*N* = 211)

Identified issues	*N* ^a^	%
Patient’s social, economic and/or administrative problems	100	51.3
Patient/provider differences in illness-related beliefs	87	44.6
Language barriers	79	40.5
Patient’s mistrust	40	20.5
Untreated mental health issues	38	19.5
Patient’s low health literacy, unrealistic expectations of medicine	37	19.0
Severe medical condition or poor prognosis	29	14.9
Clinicians’ disbelief, prejudice towards patient	20	10.3
Patient/provider conflict regarding illness management	20	10.3
Trauma and loss	17	7.5
Institutional barriers (changes of health care providers, hospital visiting policy, etc.)	15	7.7
Complex or unfamiliar family dynamics	13	6.7
Other	50	25.6

^a^The sum of requests is greater than 211 because several issues were identified for each referral

The most frequently identified problems related to the patient’s social, economic and administrative situation. In slightly over half the cases, patients had financial, housing or permit problems that compromised illness management either by materially limiting their ability to adhere to treatment or because patients gave priority to problems other than their health problems. Effective communication was often hindered by undetected, underestimated or unaddressed patient/provider differences in illness-related beliefs, language proficiency and health literacy, as well by the medical complexity of the patient’s condition. In some cases, prejudice and mistrust on the part of clinicians and/or patients contributed to a poor therapeutic alliance. Many patients were also found to be suffering from mental health problems, often related to their migration history and precarious social and administrative status.

Table 9 provides examples of key issues and recommendations based on the consultant’s assessment.

**Table 9:** Examples of key issues identified during patient cultural assessment and main recommendations issued

Brief case description	Issues identified during cultural assessment	Main recommendations
A. Young recent immigrant female patient treated for sarcoidosis with major side effects, isolated and depressed.	• Language barrier: the patient’s younger sister usually translated. The patient was somewhat reluctant to talk openly in front of her sister for fear she would tell others, and the sister did not effectively translate all that was said. • Cultural meaning of the illness: the patient and her mother (the only other family member who was aware of her disease) feared that knowledge of her disease would ruin her opportunities to marry. In addition, she was physically unable to fulfill the important role of oldest daughter, which caused tensions at home.	• Use a professional interpreter to allow the patient to freely express her feelings and concerns. • Discuss and distinguish between the side effects of treatment and the symptoms of illness. • Try to destigmatize her illness by reassuring the patient that she can live a normal life even with sarcoidosis.
B. Female visible minority patient in her late twenties, hospitalized for an acute abdominal infection refusing care.	• Language barrier: No local interpreter was available that spoke the patient’s language. Communication with her doctors and nurses was in English, but neither the patient nor many of her health care providers spoke it fluently. • Mistrust: The patient mistrusted the hospital because she developed an abdominal infection after an initial laparoscopy. • Beliefs about the body: The patient believes the blood draws and antibiotics are “drying her out” and making her weak. Lack of knowledge about the internal workings of the body and medicine in general make it difficult for the patient to understand the doctors’ explanations of her disease and its treatment.	• Information was provided on a telephone interpreting service that had interpreters for the patient’s language. • Make time to meet with the patient, answer her questions and concerns, restore trust and find common ground. • Use simple language and drawings to address the origins of her infection, the reason for frequent blood tests, how the body replaces blood, the anatomy of the stomach and purpose of the drain. • Address the concerns of the patient and her family about the proposed surgery.
C. Middle aged ex-refugee male patient with obsessive-compulsive disorder and chronic pain	• Multiple losses and trauma: the patients’ narrative reveals a succession of social and economical losses, traumatic experiences and a strong feeling of shame and injustice that was left unrecognized by public services (law, disability pension). • Precarious situation: lack of financial means, unemployment, inadequate housing and lack of access to social services • Explanatory model: his karmic explanation of misfortune was culturally congruent, but his compulsive thoughts of wrong-doing seemed more likely to be a manifestation of psychiatric illness. He firmly believed traditional medicine from his homeland could help him, as it had done so in the past.	• Refer patient to social-legal services to help with workplace accident compensation • Maintain a combination of cognitive behavioral therapy and physical therapy sessions • Encourage counseling with the Buddhist monk (who had served as cultural informant) • Explore feasibility of prolonged visit to home country for traditional treatment.

### Recommendations made by the CCS to referring clinicians

In addition to providing clinicians with detailed information about their patients’ illness-related beliefs, expectations and concerns, consultants made specific recommendations to referring clinicians in 211 cases. These generally involved adapting communication strategies to patients’ needs (using an interpreter, simplifying explanations, taking time to discuss patients’ concerns) and collaborating with other health and social resources (Table 10).

**Table 10:** Recommendations made by the Cultural consultation

Recommendation	*N* ^a^	%
Strategies to improve communication/understanding		
Modify communication style (simplify language, avoid jargon, use simple images or metaphors, etc.)	77	36.7
Use an interpreter	72	34.3
Explore/take into account the patient’s social situation	62	29.5
Explore/take into account patient opinion/preferences	25	12
Involve others in patient care		
Refer to mental health services	50	23.8
Refer to social services	50	23.8
Refer to other (non mental health) professional (GP, physical therapist)	46	21.9
Refer to specific cultural/religious resources (imam, community association, traditional healer, etc.)	37	17.5
Include family/relatives in patient management	34	16.2
Modify illness management or treatment plan	48	22.9
Other	58	27.6

^a^Recommendations were emitted for 211 cases. For some cases, more than one recommendation was given

Language barriers and patients’ low health literacy were often at the root of communication problems and by bringing in an interpreter or helping the clinician to adapt their explanations to patients’ level of knowledge, difficulties and frustrations could be attenuated. Recognizing the impact of social and economic difficulties on patients’ ability to adhere to treatment recommendations also helped clinicians to empathize and look for ways to adapt to patients’ needs.

### Clinicians’ perspectives regarding the CCS

We interviewed 51 of the 76 clinicians who had requested a consultation between March 2006 and October 2008, and between September 2009 and October 2011. These included 43 physicians (23 interns, 13 chief residents, 5 attendings, and 2 private physicians), 2 nurses and 6 social workers. Twenty-five clinicians declined to participate because they had either left the institution, were too busy or were unavailable because they were on vacation or parental leave.

#### Overall satisfaction with the CCS

Overall, clinicians were very satisfied with the CC. Forty-seven clinicians (92%) rated their satisfaction as 5 or 6 on a scale of 1–6, and all clinicians said they would call upon the CCS again in the future and recommend it to a colleague. However, only 36 (71%) gave a score of 5 or 6 when asked how useful the CC had been for the particular clinical case.

When asked about the discrepancy between their high level of satisfaction but lower score on usefulness, several clinicians explained that even in situations where there was no easy solution to the clinical problem motivating the CC request, a better understanding of their patient’s predicament helped them to tolerate the situation and regain empathy for the patient. A typical example of this was when a patient refused a treatment or medical procedure that the clinician considered vital. The CC helped them understand the reasons behind the refusal, reduce their sense of frustration and better relate to the patient. Thus they were very satisfied with the consultation but felt it was only moderately useful because the patient had not changed his mind after the CC.

#### How the cultural consultation helped clinicians

A large majority of clinicians reported that the CC helped them to better understand the patient’s illness-related ideas and expectations and the ways in which social and cultural factors were affecting the patient and the patient’s care. Over half said the CC helped them to communicate more effectively with their patients (Table 11).

*The CC untied a knot…*

*The CC helped create a trusting relationship between the patient and the medical team that was beneficial to further treatment.*

*I better understood the patient’s story; I changed my way of communicating and even used other words with him afterwards.*

Over half of clinicians also said it was helpful to receive general information that could be useful beyond the specific patient that motivated their CC request. This included information about immigration and asylum (56.9%; e.g. types of residence permits, health insurance coverage, administrative procedures and living/working conditions), as well as about social, legal or community services that were available to immigrant patients (54.9%).

**Table 11:** Clinicians’ perceptions of how the Cultural consultation service helped (*N* = 51)

Ways in which the CCS helped clinicians	*N* ^a^	%
Better understand how social and cultural factors affect the case	46	90.2
Better understand the patient’s illness-related ideas and expectations	38	74.5
Communicate more effectively with the patient	30	58.8
Learn about community resources available for immigrant patients	29	56.9
Better understand asylum and/or immigration related issues	28	54.9
Improve the patient’s adherence to treatment	20	39.2
Clarify the patient’s treatment plan	20	39.2
Clarify the patient’s diagnosis	9	17.6

^a^The total n is greater than 51 because clinicians could check more than one answer

#### What clinicians appreciated about the CCS

88.5% of clinicians considered the social and cultural expertise brought by the CCS to be very or somewhat important, and 76.5% appreciated having a space to discuss complex cases and receive an external perspective (72.5%). They expressed feelings of relief and reassurance after hearing an external and non-judgmental view of a complex situation.

*It did us a lot of good that someone confirmed we were on the right track. We felt relieved and not at fault any more.*

*The CC helped me feel less guilty, take a step back and review my evaluation of the family and my objectives of care for this patient.*

In spontaneous comments, some clinicians also explained that they didn’t have the time or skills necessary to explore cultural factors affecting care, felt frustrated or at an impasse in a complex clinical case.

Others said they appreciated being able to observe the consultant conduct a clinical ethnography interview because it provided them with a new approach for communicating with their patients.

*The CC gave me tools that I can apply to similar situations in the future.*

*This experience will help me for the rest of my career. The CC opened a door to new aspects of the relationship one can have with a patient.*

#### Suggestions for improving the CCS

Finally, we asked clinicians for suggestions on how we might improve the CCS. Their recommendations included making the CCS more visible institutionally through the hospital website and during continuing education activities; creating more opportunities for health care teams to meet and discuss complex cases with the CCS; and by making the CC assessment reports more visible in the patient’s electronic file. Several clinicians would have liked the CCS to take over the patient’s care altogether or provide systematic follow-up on these cases.

## Discussion

Requests for cultural consultations are relatively few in number but tend to involve patient care situations with complex social, cultural and medical issues. Based on what we have learned from previous surveys at our hospital, clinicians deal daily with the challenges of providing care across language, social and cultural differences [32, 41], and they may not feel the need for outside support in the majority of these situations.

A number of studies have found that clinicians are most challenged when complex medical issues are accompanied by language barriers, social problems they feel powerless to address, and unfamiliar cultural norms and practices [4, 42]. Although such difficulties are not specific to immigrant patients, to date we have only received CC requests for situations involving patients of migrant background. In these situations clinicians have tended to “culturalize” their difficulties; that is, they focused on the patient’s culture as the main cause of their difficulties, rather than consider the role of socioeconomic and institutional barriers, or medical culture itself [24, 43, 44]. Grove and Zwi argue that this process of “othering” helps to secure one’s identity while distancing those who deviate from the dominant norm [44]. In our sample, the most common reason to refer to the CCS is “lack of adherence”, a common clinical problem regardless of patient origin. This may suggest that clinicians faced with a difficult situation where the patient does not behave as expected within the “culture of medicine” unintentionally resort to a process of distancing the patient as a cultural other, thus reinforcing their professional identity [45].

Furthermore, European and Swiss efforts at managing diversity in health care may have inadvertently contributed to this tendency to “culturalize” immigrant patients through their focus on developing “Migrant Friendly Hospitals” (MFHs) [45, 46]. More recently, there have been calls to broaden the focus of cultural competence efforts to ensure effective communication and quality care for all patients, not just migrants. In 2014, the Swiss MFH network changed its name to Swiss Hospitals for Equity to reflect this evolution [47].

Clinical ethnography can contribute to greater awareness of the role of medical culture in patient/provider communication difficulties, regardless of the patient’s origin [48–50]. It is often said that one of the central tasks of anthropology is “to make the strange familiar and the familiar strange,” [51] and in the context of our CCS, clinical ethnography allows us to “complete the patient’s story” and put behavior in context. Understanding the patient’s reality allows the clinician to “make sense” of what appears initially to be strange or illogical behavior, and to have more empathy for the patient.

Despite the existence of several pre and post-graduate teaching activities at our hospital that address the importance of identifying the social and cultural factors affecting care [52], there are few opportunities for clinicians to observe how clinical ethnographic interviewing is done and to experience its usefulness for patient care. We believe that the CCS provides such an opportunity, and can contribute to integrating such practices into clinical care. A study in London that combined classroom training in cultural competence with ‘in vivo’ training through a similar cultural consultation service found that clinical staff deepened their understanding of the importance of assessing the social and cultural factors affecting care, and learned to use a narrative, ethnographic approach with patients [53].

### Limitations

The most important limitation of our evaluation is that we are unable to say whether contact with the CCS has led to more or better clinical ethnographic interviewing on the part of clinicians. Clinicians appear to benefit from the information we gather during patient assessments, and broaden their views of the social and cultural contexts of patient care, but do they then explore these factors on their own with future patients? In the face of chronic time constraints, and their appreciation of the opportunity to discuss cases with us, they may be more inclined to call the CCS the next time they encounter difficulties due to social and cultural differences, rather than attempt a clinical ethnography interview on their own. In order to change practices, it may be necessary to directly link the CCS experience with more purposive teaching, as in the London model described above.

The retrospective, descriptive analysis of our cultural consultation service was based on written records of the consultation requests (intake forms and patient assessment reports). These records contain factual data, but they also reflect the consultants’ interpretation of the social and cultural issues affecting communication and care. We regularly discussed cases with our expanded CCS team in order to bring in other perspectives, but different interpretations and recommendations might have been generated by other consultants. In addition, our intake forms and reports were not always complete and certain patient data were missing. With regards to clinicians’ satisfaction with the CCS, the views expressed by our sample may not be representative of all clinicians who contacted the CCS.

Despite these limitations, we feel that these data allowed us to see some general trends with respect to the issues and situations that pose challenges for clinicians, and to identify the kinds of support and information that may help them to care more effectively for socially culturally diverse patients.

## Conclusion

Our experience suggests that a hospital-based service that provides direct support and role modeling of culturally sensitive interviewing to clinicians can contribute to better patient/provider communication and understanding. A cultural consultation service such as ours can contribute to institutional cultural competence by drawing attention to the challenges of caring for diverse patient populations, identifying the training needs of clinicians and gaps in resource provision, and providing hands-on experience with clinical ethnographic interviewing.

## Abbreviations

CC: Cultural consultation
CCS: Cultural consultation service
CF: Cultural formulation
HUG: Hôpitaux Universitaires de Genève (Geneva University Hospitals)
MFH: Migrant Friendly Hospitals
SPSS: Statistical package for the social science

## Footnotes

1 During the first years of the CCS, a small number of assessments were conducted by a migrant care nurse specialist.
